# Serine protease inhibitor Kazal type 1 (SPINK1) promotes proliferation, migration, invasion and radiation resistance in rectal cancer patients receiving concurrent chemoradiotherapy: a potential target for precision medicine

**DOI:** 10.1007/s13577-022-00776-4

**Published:** 2022-09-02

**Authors:** Yi-Ting Chen, Tzu-Ting Tseng, Hung-Pei Tsai, Shih-Hsun Kuo, Ming-Yii Huang, Jaw-Yuan Wang, Chee-Yin Chai

**Affiliations:** 1 Department of Pathology, Kaohsiung Medical University Hospital, Kaohsiung Medical University, No. 100, Tzyou 1st Road, Kaohsiung, 807 Taiwan; 2 Department of Pathology, Faculty of Medicine, College of Medicine, Kaohsiung Medical University, Kaohsiung, Taiwan; 3 Graduate Institute of Medicine, College of Medicine, Kaohsiung Medical University, Kaohsiung, Taiwan; 4 Division of Neurosurgery, Department of Surgery, Kaohsiung Medical University Hospital, Kaohsiung, Taiwan; 5 Department of Radiation Oncology, Kaohsiung Medical University Hospital, Kaohsiung Medical University, Kaohsiung, Taiwan; 6 Department of Radiation Oncology, Faculty of Medicine, College of Medicine, Kaohsiung Medical University, Kaohsiung, Taiwan; 7 Division of Colorectal Surgery, Department of Surgery, Kaohsiung Medical University Hospital, Kaohsiung Medical University, Kaohsiung, Taiwan; 8 Department of Surgery, Faculty of Medicine, College of Medicine, Kaohsiung Medical University, Kaohsiung, Taiwan; 9 Graduate Institute of Clinical Medicine, College of Medicine, Kaohsiung Medical University, Kaohsiung, Taiwan; 10 Center for Cancer Research, Kaohsiung Medical University, Kaohsiung, Taiwan; 11 Institute of Biomedical Sciences, National Sun Yat-Sen University, Kaohsiung, Taiwan

**Keywords:** Serine peptidase inhibitor Kazal type-1, Concurrent chemoradiotherapy, Rectal cancer, Immunohistochemical staining, Prognostic marker

## Abstract

Serine peptidase inhibitor Kazal type-1 (SPINK1), a trypsin kinase inhibitor, is known to be associated with inflammation and pathogenesis. The aim in this study was to demonstrate the clinicopathological role and progression of SPINK1 in rectal cancer (RC) patients undergoing concurrent chemoradiotherapy (CCRT). Immunohistochemical staining for SPINK1 protein expression in 111 RC cases revealed high SPINK1 expression was significantly associated with perineural invasion and poor CCRT response in pre-CCRT specimens. In addition, multivariable analyses showed that pre-CCRT SPINK1 expression was a significant prognostic marker of both overall and disease-free survival in RC patients receiving pre-operative CCRT; furthermore, in vitro studies demonstrated SPINK1 interacted with EGFR to promote the abilities of proliferation, migration and invasion attenuated by SPINK1 si-RNA via ERK, p38, and JNK pathways. SPINK1 was also found to regulate radio-resistance in CRC cell lines. In conclusion, SPINK1 expression is an independent prognostic marker in patients receiving pre-operative CCRT, and SPINK1 regulates proliferation, migration and invasion via EGFR-downstream ERK, p38 and JNK pathways. The phenotypes of radiosensitivity that could be reversed with attenuation of SPINK1 levels suggest that targeting SPINK1 might offer a strategy for optimal precision medicine.

## Introduction

Colorectal cancer (CRC) is the third most commonly diagnosed cancer in men and the second in women worldwide. About 1.9 million newly diagnosed CRC cases were reported leading to 935,000 deaths in 2020 [1]. Increased physical exercise, avoidance of a typical western sedentary lifestyle, reduction of intake of alcohol and consumption of tobacco might all reduce risk of CRC [2]. Regular colorectal screening is also known to decrease cancer-associated mortality in high-income countries. Currently, traditional surgical intervention combined with chemoradiotherapy and targeted therapy play important therapeutic roles for precision medicine.

Serine protease inhibitor Kazal-type 1 (SPINK1) was first discovered in the urine of an ovarian cancer patient and then isolated from pancreatic acinar cells by Kazal et al. [3]. The main function of this trypsin kinase inhibitor is to prevent autodigestion or proteolysis of normal pancreatic glands, which is associated with inflammation and cancer. Recent studies have shown that SPINK1 has an important prognostic role in development of many cancers and could be targeted therapeutically [4]. In our previous investigation, the prognostic role of SPINK1 in general colon cancer was studied in vivo, revealing that high SPINK1 expression was associated with advanced cancer stage and poor prognosis [5]. Increased liver metastasis has also been noted in CRC patients with higher SPINK1 expression [6].

The molecular structure of SPINK1 has been identified as being similar to the epidermal growth factor (EGF), so SPINK1 may be regarded as having a growth factor effect by binding to the epidermal growth factor receptor (EGFR) pathway [7]. EGFR is a cell-surface receptor and regulates intracellular messenger transduction, and once activated, it can induce tyrosine autophosphorylation while causing cell proliferation, inhibition of apoptosis and angiogenesis [8]. Previous studies have shown co-expression of SPINK1 and EGFR in pancreatic tumors [9] and SPINK1 promote pancreatic cancer cells through the mitogen-activated protein kinase (MAPK) pathway in vitro [10]. In prostate cancer, SPINK1 is also mediated by EGFR and is incidentally related to aggressive disease in patients, so it has been used as a plausible target therapy in prostate cancer [11]. However, the pathogenesis of SPINK1 in CRC in vitro needs to be explored.

For advanced rectal cancer (RC) patients, pre-operative neoadjuvant concurrent chemoradiotherapy (CCRT) is the treatment of choice in addition to surgery. Radiation can induce oxidative damage, apoptosis and affect the tumor microenvironment by remolding the extracellular matrix [12]. SPINK1, as a trypsin inhibitor, might also be involved in progression of invasion through tumor-extracellular matrix interaction. The radio-sensitizing effect and pre- and post-CCRT expression in prognostic roles of SPINK1 are still to be determine.

The aim of this study was to evaluate the use of SPINK1 as a prognostic indicator in pre- and post-CCRT tissue in RC patients. Also, the pathogenesis of SPINK1 in CRC was investigated in vitro. To our knowledge, this is the first study performing immunohistochemical (IHC) and molecular analysis to explore the prognostic value of SPINK1 expression combined with CCRT response status in RC patients.

## Materials and methods

### Samples

The study was approved by the Institutional Review Board of Kaohsiung Medical University Hospital (KMUHIRB-E(I)-20160145). Overall, the study included 111 cases of advanced RC receiving pre-operative care that had total meso-rectal resection for tumor excision and lymph node dissection at Kaohsiung Medical University Hospital from 2010 to 2014. Patients with pre-operative distant metastasis, synchronous or metachronous malignancy, complete remission after CCRT or who were without complete medical records were excluded, as were 19 patients lacking both pre- and post-CCRT specimens for analysis.

All patients received colonoscopy and an abdominopelvic computed tomography image study to investigate the clinical stage according to the American Joint Committee on Cancer (AJCC) 8th edition staging system initially [13]. Patients with clinical T3–4 or N1–2 received preoperative CCRT [14] and the operative procedures were defined according to published literature [15].

### Pathologic evaluation

All specimens including pre-CCRT biopsy and post-CCRT surgical tissues were processed according to standard pathologic protocols. The hematoxylin and eosin staining results were reviewed to confirm the diagnosis and pathologic features, including tumor grade, invasion status (lymphovascular or perineural), and disease stage [13]. Tumor regression score (TRS) was also analyzed to evaluate the response of CCRT, according to the grading system of College of American Pathologists. A four-grade scale was recommended and divided into grade 0 (complete response), grade 1 (moderate response), grade 2 (minimal response) and grade 3 (poor response) [16].

### IHC staining and scoring

Tissue samples of biopsy and surgical specimens were cut into thicknesses of 4 μm, fixed in formalin and embedded in paraffin, with sections then being dried, deparaffinized and finally rehydrated. Heat-mediated antigen retrieval was used by boiling under pressure in Target Retrieval Buffer (pH 9.0; Dako, Glostrup, Denmark) for 8 min. The slides were washed with Tris buffer solution after using 3% hydrogen peroxide to block endogenous peroxidase activity for 5 min at room temperature. The immunohistochemical study was performed using anti-SPINK1 (1:800, Abcam, USA, #ab58227) as the primary antibody with positive and negative controls included for quality control. Semi-quantitative analysis of SPINK1 expression was performed independently by two pathologists.

Expression of SPINK1 was recorded in terms of the percentage of tumor cells with cytoplasmic staining as described in previous studies [6, 17, 18]. Highest immunoreactivity values of the cancer cells were recorded, with more than 50% immunoreactivity of SPINK1 in tumor cells was regarded as high expression. In cases with discrepancy, both pathologists re-analyzed the IHC slide together and a final score was made by consensus.

### Cell culture, transfection siRNA and recombinant plasmid

SW48, SW480, COLO205, SW620 and HCT-116 cells were purchased from Bioresource Collection and Research Center (Taiwan), and HT-29 cells were from American Type Culture Collection (USA) Cell Line Bank. SW48 and SW480 were cultured with L15 medium including 10% FBS. COLO205 was cultured with RPMI medium including 10% FBS. HT-29 was cultured with McCoy’s 5A medium (Gibco; USA; 16,600-082) including 10% FBS. SW620 and HCT-116 cells were cultured with McCoy’s 5A medium including 10% FBS. All cell lines were seeded at 1 × 10^5^ cells per 24-well plate and grown at 37 °C with 5% CO_2_. Cell numbers were determined using a hemocytometer.

SPINK1 siRNA in colorectal cells was achieved using DharmaFECT™ siRNA transfection Reagents (horizon). The sequences of human SPINK1 siRNA#1 and 2 were 5´-CAAUGUUACAAUGAACUUdTdT-3´ and 5´-AAGUUCAUUGUAACAUUUGdTdT-3´. 5 μM SPINK1 siRNA was used through transfection. Following transfection with siRNA, cells were cultured for 3 days before use, then SPINK1 protein expression was detected by western blot analysis. Negative control siRNA (sense: 5´-UUCUCCGAACGUGUCACGUTT-3´ and antisense: 5´-ACGUGACACGUUCGGAGAATT-3´) was also used.

SPINK1-overexpressing transient cells with a minigene composed of the CAG promoter were then coupled to the human *SPINK1* gene. A total of 50 nmol/L DNA was transfected using Lipofectamine 3000 (Invitrogen). The cells were incubated at 37 °C for 48 h and were subsequently harvested for the following studies. SPINK1 protein expression was detected by western blot analysis.

### Protein extraction and western blot

All samples were prepared in 100 μl RIPA lysis buffer. 30 μg protein of every sample was loaded in the wells of SDS-PAGE with 80 V for 2 h then samples were transferred from the gel to the PVDF membrane with 400 mA for a further 2 h. After 1 h use of blocking buffer, the membranes were incubated with primary antibody SPINK1 (1:500; Abcam, USA, #ab58227), p-p38 (Cell Signaling, #9211), p38 (Cell Signaling, #9212), p-JNK (Cell Signaling, #4668), JNK (Cell Signaling, #9252), p-ERK (Cell Signaling, #9101), ERK (Cell Signaling, #9102), p-Akt (Cell Signaling, #9271S), AKT (Cell Signaling, #9272S), Stat3 (Santa Cruz, #sc-8019), p-PI3K (ABGENT, AP50389), PI3K (ABGENT, AP52796), β-actin (Sigma-Aldrich, MAB1501R), and secondary antibody Goat anti-Rabbit (1:2000; Millipore; AP132P) or Goat anti-Mouse (1:2000; Millipore; AP124P)] was applied. The signals were detected by ECL solution (Western Lightning; 205-14,621) with MINICHEMI (Thermo). Intensity of Western blot bands was digitally analyzed using ImageJ software.

### Cell viability

The cell lines were obtained by dissolving the cells in McCoy’s 5A (HT-29) and DMEM (HCT116) culture medium containing 10% serum and then placed in a 24-well plate with about 3000 cells of 500 μl volume in each well. These treated cells were incubated under the condition of 5% CO_2_, saturated humidity, and 37 °C for 24 h. Viable cell counts were obtained following MTT assay after treating with negative siRNA, SPINK1 siRNA, p-SPINK1 or inhibitors for 24, 48 and 72 h.

### Migration and invasion assays in vitro

Cell migration assay was used through wound-healing assay (ibid: 80,209). The average width of the wounded gaps was evaluated from the two sides of the wound. Wound healing assay for 24-well plates was coated with 3 × 10^5^ cells and cultured at 37 °C for 12 h and then either siRNA, p-SPINK1 or inhibitor was added. After 1 day, samples were washed twice with PBS, and pictures were taken at 16, 24, 36 and 48 h. Cell invasion assay was performed using a Transwell (CORNING; COR3452) in vitro. Incubated cells with siRNA, p-SPINK1 or inhibitors were seeded at 5 × 10^3^ per insert and the lower chamber of the Transwell was filled with 0.500 ml medium including 10% FBS. After 24 h, cells remaining on the upper surface of the Transwell membrane were removed by cotton swab. Cells that had invaded through the Transwell to the bottom of the insert were fixed by formalin, stained by 0.5% crystal violet, photographed and quantified by counting them in six random high-powered fields.

### Inhibitor assay

AG-490 (# S1143), a specific inhibitor of the tyrosine kinase activity of the EGFR, was purchased from Selleckchem; U0126 (#1668–5, MEK inhibitor) was from BioVision; SB203580 (#HY-10256), a p38 inhibitor, and SB600125 (# HY-12041), a JNK inhibitor, were purchased from MEC; while four specific inhibitors, AG-490 (40 μM, EGFR inhibitor), U0126 (10 μM, MEK inhibitor), SB203580 (10 μM, p38 inhibitor) and SB600125 (10 μM, JNK inhibitor) were used in these studies.

### Immunofluorescent staining

The formalin-fixed and paraffin-embedded tissue slides were cut into thicknesses of 4 μm then deparaffinized, and finally rehydrated. Heat-mediated antigen retrieval was used by boiling under pressure in Target Retrieval Buffer (pH 9.0; Dako, Glostrup, Denmark) for 30 min. Anti-SPINK1 primary antibody was used for detection along with the corresponding red dye-labelled secondary antibody (20,901, goat anti-mouse IgG, LEADGENE, Taiwan). Anti-EGFR primary antibody was used for detection along with the corresponding green dye-labelled secondary antibody (C04013, goat anti-rabbit IgG, Croyez, Taiwan). Afterwards, the slides were washed three times with PBS and sealed with DAPI (Sigma, F6057)-containing mounting medium. The slides were viewed under a fluorescence microscope.

### Co-immunoprecipitation

Cells were chilled to 4 °C for 10 min and cell extracts prepared with lysis buffer as described above. Lysates were cleared by centrifugation at 13,200 rpm for 30 min and incubated with anti-EGFR (1:1000; Merck; #05–1047) or IgG (1:5000; proteintech; 10,283–1-AP) antibody covalently in 4 °C for overnight. Beads were then added for 30 min and then extensively washed in lysis buffer and then removed supernatant with Magdorf. Sample including beads was washed using washing buffer and added 60 ul of 0.2 M Glycine–HCl, pH 2.5 for 2 min at room temperature. Finally, transfer the supernatant to a new tube and added 5 ul of 1 M Tri-HCl, pH 8.5. This sample was detected using immunoblotting. The proteins were separated on a 10% SDS–polyacrylamide gel with 70 V for 3 h, transferred to a PVDF membrane with 400 mA for 2 h, and analyzed by immunoblotting with the EGFR (1:2000; proteintech; 18,986–1-AP), SPINK1 (1:500; abcam; ab58227) and IgG (1:10,000; proteintech; 30,000–0-AP) antibody.

### Ionizing irradiation exposure and clonogenic assay

Treated or non-treated control cells were exposed to gamma rays (ELEKTA, Axesse) at a single dosage of 0, 1, 2, 4, 8 Gy. After irradiation, the cells were seeded onto 6 cm dishes and incubated for 14 days without disturbance. The seeded cell number increased as radiation dosage increased. The culture medium was replaced with fresh medium every 2 days. Formed colonies were visualized by staining with 0.02% crystal violet solution (*w*/*v* in 75% ethanol) and imaged using a scanner. The plating efficiency was determined as the ratio of the number of colonies divided by the number of cells seeded, while the surviving fraction was determined from the ratio of plating efficacy of irradiation cells compared to the non-irradiated control.

### Statistical analysis

All statistical analyses were performed with SPSS 19.0 (Chicago, IL, USA). Chi-square test was used to determine SPINK1 protein expression IHC study in compared pre- and post-CCRT specimens correlated with clinicopathological factors. Overall survival (OS) was measured from the date of surgery to the date of death. Disease-free survival (DFS) was defined as the duration between date of primary treatment to recurrence, metastasis or to the last follow-up date. The survival rate was analyzed by Kaplan–Meier method with log-rank test while multivariate Cox regression analyses were used to verify the independent effect of each parameter considered. All tests were 2-sided, and a *p* value less than 0.05 was considered statistically significant.

## Results

### SPINK1 expression in pre- and post-CCRT rectal carcinoma specimens

**Fig. 1:**
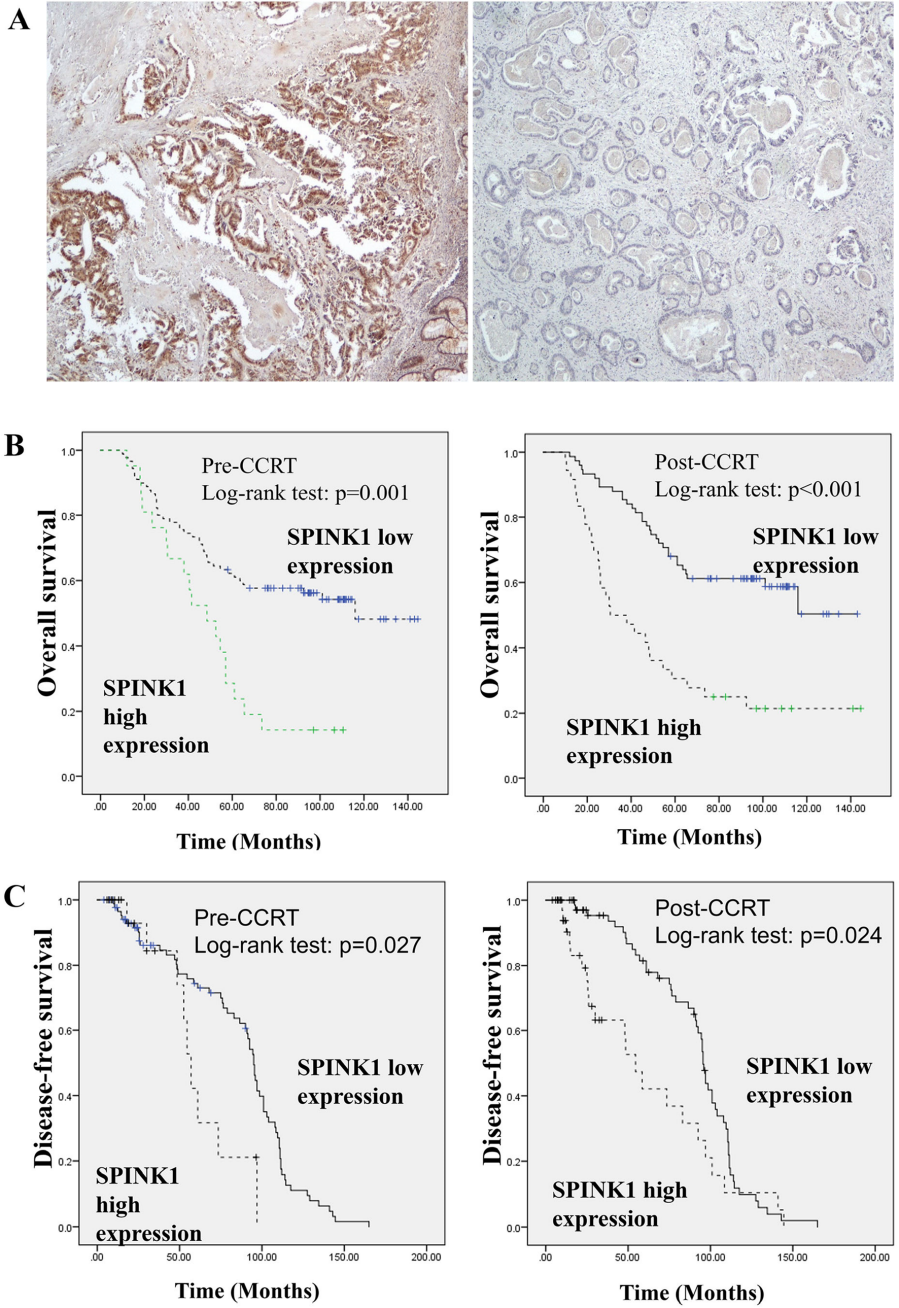
Representative IHC staining of SPINK1 in RC specimens. High and low (**A**). Kaplan–Meier plots of OS (**B**) and DFS (**C**) estimated in 111 RC cases receiving pre-operative CCRT by pre-CCRT and post-CCRT SPINK1 expression. High SPINK1 expression was significantly associated with worse OS and DFS

Table 1 presents the expressions of pre-CCRT SPINK1 in relation to RC and patient characteristics. The immunostaining results revealed high SPINK1 expression in 21 (18.9%) of 111 pre-CCRT RC cases (Fig. 1A).

**Table 1:** Correlation of pre-CCRT SPINK1 expression with clinicopathological parameters in RC patients

Variables	*n*	SPINK1 expression		
		High	Low	*p* value
Total	111	21 (18.9%)	90 (80.1%)	
Age (y/o)				0.804
≦ 75	69 (62.2%)	14 (20%)	55 (80%)	
> 75	42 (37.8%)	7 (16.7%)	35 (83.3%)	
Gender				0.078
Male	69 (62.2%)	17 (24.6%)	52 (75.4%)	
Female	42 (37.8%)	4 (9.5%)	38 (90.5%)	
Tumor grade				0.123
G1 and G2	104 (93.7%)	18 (17.3%)	86 (82.7%)	
G3	7 (6.3%)	3 (42.9%)	4 (57.1%)	
Lymph-vascular invasion				1.000
Negative	86 (77.4%)	16 (18.6%)	70 (81.4%)	
Positive	25 (22.6%)	5 (20%)	20 (80%)	
Perineural invasion				0.028*
Negative	81 (73%)	11 (13.6%)	70 (86.4%)	
Positive	30 (27%)	10 (33.3%)	20 (66.7%)	
Tumor stage (ypT)				0.334
ypT0–2	47 (42.3%)	11 (23.4%)	36 (76.4%)	
ypT3–4	64 (57.7%)	10 (15.6%)	54 (85.4%)	
Lymph node stage (ypN)				0.799
ypN0	73 (65.8%)	13 (17.8%)	60 (82.2%)	
ypN1–2	38 (34.2%)	8 (21.1%)	30 (78.9%)	
Metastasis status (ypM)				0.264
No	98 (88.2%)	17 (17.3%)	81 (82.7%)	
Yes	13 (11.8%)	4 (30.8%)	9 (69.2%)	
Disease stage				0.327
Early (I and II)	69 (62.2%)	11 (15.9%)	58 (84.1%)	
Late (III and IV)	42 (37.8%)	10 (23.8%)	32 (76.2%)	
Tumor regression score				0.006*
0–1	46 (41.4%)	3 (6.5%)	43 (93.5%)	
2–3	65 (58.6%)	18 (27.7%)	47 (72.3%)	

*Statistically significant (*p* < 0.05)

Immunoreactivity of SPINK1 significantly differed by peri-neural invasion (PNI) (*p* = 0.028). Almost half of the SPINK1 high-expression patients had PNI; and high SPINK1 was significantly associated with high TRS, which indicated poor response to CCRT (*p* = 0.006).

Table 2 shows the expressions of post-CCRT SPINK1 with the IHC results finding high SPINK1 expression in 36 (32.4%) of 111 post-CCRT RC cases. Immunoreactivity of post-SPINK1 significantly differed by tumor stage (*p* = 0.040), lymph node stage (*p* = 0.019), metastasis status (*p* = 0.026) and disease stage (*p* = 0.036). High SPINK1 expression was significantly associated with advanced cancer stage. Moreover, patients with high post-CCRT SPINK1 expression had significantly higher TRS (*p* = 0.001).

**Table 2:** Correlation of post-CCRT SPINK1 expression with clinicopathological parameters in RC patients

Variables	*n*	SPINK1 expression		
		High	Low	*p* value
Total	111	36(32.4%)	75(67.6%)	
Age (y/o)				0.676
≦ 75	69 (62.2%)	21 (30.4%)	48 (69.6%)	
> 75	42 (37.8%)	15 (35.7%)	27 (64.3%)	
Gender				0.303
Male	69 (62.2%)	25 (36.2%)	44 (63.8%)	
Female	42 (37.8%)	11 (26.2%)	31 (73.8%)	
Tumor grade				1.000
G1 and G2	104 (93.7%)	34 (32.7%)	70 (67.3%)	
G3	7 (6.3%)	2 (28.6%)	5 (71.4%)	
Lymph-vascular invasion				0.809
Negative	86 (77.4%)	27 (31.4%)	59 (68.6%)	
Positive	25 (22.6%)	9 (36%)	16 (64%)	
Perineural invasion				1.000
Negative	81 (73%)	26 (32.1%)	55 (67.9%)	
Positive	30 (27%)	10 (33.3%)	20 (66.7%)	
Tumor stage (yT)				0.040*
yT0–2	47 (42.3%)	10 (21.3%)	37 (78.7%)	
yT3–4	64 (57.7%)	26 (40.6%)	48 (59.4%)	
Lymph node stage (yN)				0.019*
yN0	73 (65.8%)	18 (24.7%)	55 (75.3%)	
yN1–2	38 (34.2%)	18 (47.4%)	20 (52.6%)	
Metastasis status (yM)				0.026*
No	98 (88.2%)	28 (28.6%)	70 (71.4%)	
Yes	13 (11.8%)	8 (61.5%)	5 (38.5%)	
Disease stage				0.036*
Early (I and II)	69 (62.2%)	17 (24.6%)	52 (75.4%)	
Late (III and IV)	42 (37.8%)	19 (45.2%)	23 (54.8%)	
Tumor regression score				0.001*
0–1	46 (41.4%)	7 (15.2%)	39 (84.8%)	
2–3	65 (58.6%)	29 (44.6%)	36 (55.4%)	

*Statistically significant (*p* < 0.05)

### Relationship of SPINK1 to OS and DFS

Kaplan–Meier analysis showed that SPINK1 expression correlated with OS in both pre- and post-CCRT RC specimens (*p* = 0.001 and < 0.001, respectively) (Fig. 1B). Mean survival time was significantly shorter in patients with high SPINK1 expression compared to cases with low SPINK1 expression in pre-CCRT status. Meanwhile, patients with post-CCRT high SPINK1 expression had shorter OS compared with low SPINK1 ones. Multivariable analyses revealed significantly prognostic candidates of OS were lymph node metastasis (*p* < 0.001), distant metastasis (*p* < 0.001), pathologic cancer stage (*p* = 0.003) and pre-CCRT SPINK1 (*p* = 0.003) in RC patients (Table 3).

**Table 3:** Univariate and multivariate analysis of prognostic indicators to predict overall survival and disease-free survival for rectal cancer patients

	Overall survival				Disease-free survival			
	Univariate analysis		Multivariate analysis		Univariate		Multivariate	
Covariate	HR (95% CI)	*p* value	HR (95% CI)	*p* value	HR (95% CI)	*p* value	HR (95% CI)	*p* value
Gender (female vs. male)	0.616–1.463	0.815	–	–	0.474–1.147	0.177	–	–
Age (≦ 65 y/o vs. > 65 y/o)	0.823–1.958	0.280	–	–	0.995–2.377	0.053	–	–
Tumor Grade (G1/2 vs.G3)	0.428–2.238	0.958	–	–	0.476–2.518	0.831	–	–
LVI (negative vs. positive)	0.388–3.998	0.713	–	–	0.151–1.887	0.329	–	–
PNI (negative vs. positive)	0.573–1.723	0.983	–	–	0.618–1.846	0.813	–	–
Tumor stage (ypT) (T1-2 vs. T3)	0.874–1.220	0.705	–	–	0.870–1.209	0.763	–	–
Lymph node stage (ypN) (N0 vs. N1–2)	2.139–29.707	0.002*	2.775–24.58	< 0.001*	0.331–5.604	0.668	–	–
Metastasis status (ypM)	2.685–15.666	< 0.001*	3.166–17.255	< 0.001*	1.607–8.836	0.002*	1.765–6.643	< 0.001*
Pathologic stage (stage 0–II vs. III)	1.51–18.18	0.009*	1.862–18.868	0.003*	0.134–1.629	0.233	–	–
TRS (0–1 vs. 2–3)	0.563–1.557	0.800	–	–	0.591–1.595	0.907	–	–
Pre-CCRT SPINK1 (low vs. high)	1.074–1.937	0.015*	1.144–1.926	0.003*	1.204–2.154	0.001*	1.131–1.919	0.004*
Post-CCRT SPINK1 (low vs. high)	0.872–1.414	0.396	–	–	0.956–1.506	0.105	–	–

*CI* confidence interval, *LVI* lymphovascular invasion, *PNI* perineural invasion, *TRS* tumor regression score

*p < 0.05

Kaplan–Meier analysis identified that SPINK1 expression also correlated with DFS in both pre- and post-CCRT RC specimens (*p* = 0.027 and 0.024, respectively) (Fig. 1C). Mean survival time was also significantly longer in patients with low SPINK1 expression compared to cases with high SPINK1 expression in post-CCRT specimens. Furthermore, patients with post-CCRT low SPINK1 expression had longer DFS compared with high SPINK1 ones. Multivariable analyses demonstrated that distant metastasis (*p* < 0.001) and pre-CCRT SPINK1 (*p* = 0.004) were significant prognostic markers of DFS in RC patients (Table 3).

### SPINK1 protein expression was up-regulated in CRC cell lines

**Fig. 2:**
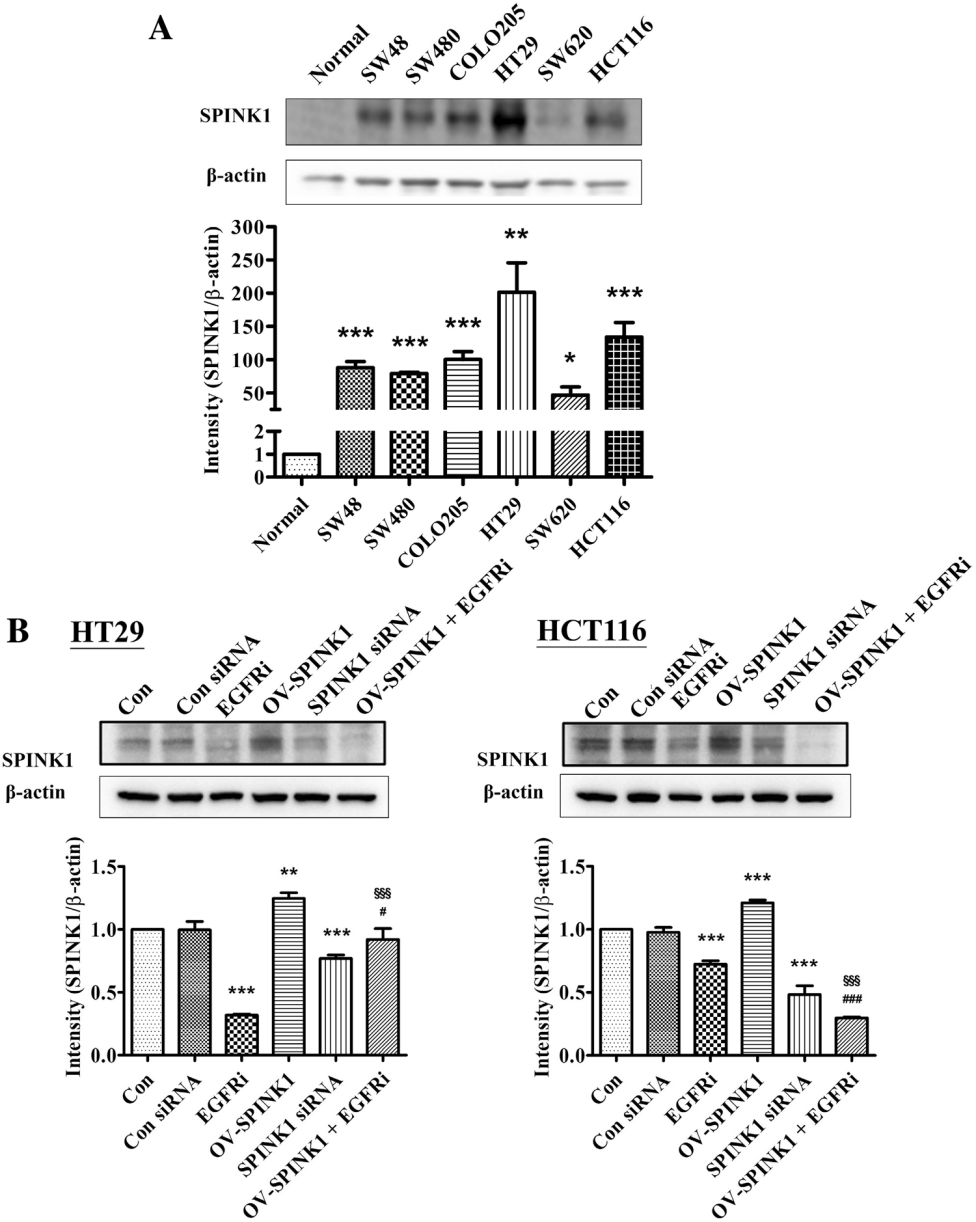
**A** Western blot of SPINK1 in all cell lines and the relative protein expressions of SPINK1 in all cell lines. **B** Western blot of SPINK1 in all cell lines and the relative protein expressions of SPINK1 in HT-29 cell lines and HCT-116 cell lines treated by non-siRNA, EGFR inhibitor (EGFRi), OV-SPINK1 and SPINK1 siRNA (with/without EGFR inhibitor). *p < 0.05, **p < 0.01 and ***p < 0.001 as compared to the control group (*n* = 3/group); ^#^*p* < 0.05, ^##^*p* < 0.01 and ^###^*p* < 0.001 as compared to the OV-SPINK1 group. ^§^*p* < 0.05, ^§§^*p* < 0.01 and ^§§§^*p* < 0.001 as compared to the EGFRi group (*n* = 3/group)

To investigate the expression levels of SPINK1 protein, western blot analysis was performed in non-neoplastic colonic mucosa and CRC cell lines SW48, SW480, COLO205, HT-29, SW620 and HCT-116. The results showed that all CRC cell lines had significantly higher expression of SPINK1 protein than normal colonic mucosa. HT-29 had the highest SPINK1 expression and HCT-116 had the second highest of all cell lines (Fig. 2A). To assay the effects of silencing of *SPINK1*, we used these two cell lines for further treatment with siRNA targeting *SPINK1* mRNA for degradation. After 72 h incubation with *SPINK1* siRNA (si-SPINK1 group) or non-siRNA (Control siRNA group), the SPINK1 protein expression was compared between control group/non-si groups and the si-SPINK1 group was analyzed by western blot analysis. In both HT-29 and HCT 116 cell lines, the results revealed that knock-down *SPINK1* down-regulated SPINK1 protein expression (*p* < 0.001) (Fig. 2B). To assay the effects of overexpression of *SPINK1* (OV-SPINK1) on cells, cells were transiently transfected with plasmid *SPINK1* expression vector (p-SPINK1). Compared with the control group, both HT-29 and HCT-116 cell lines infected by p-SPINK1 had significantly increased SPINK1 protein expression (*p* < 0.01) (Fig. 2B).

To detect the role of EGFR mediation of SPINK1 in CRC cells, HT-29 and HCT-116 cells that overexpressed SPINK1 were treated with EGFR inhibitor. Results showed that EGFR inhibitor could decrease the expression of SPINK1 protein in CRC cells with overexpressed SPINK1. Furthermore, the effect of EGFR inhibitor was reversed by hyper-endogenous SPINK1 (Fig. 2B).

These data showed that higher SPINK1 protein expression was noted in CRC cells. Hyper-expression or lower-expression of SPINK1 protein could be modified by p-SPINK1 or also by adding siRNA or EGFR inhibitor, also indicating interaction between SPINK1 and EGFR.

### SPINK1 plays a role in proliferation of CRC cell lines

**Fig. 3:**
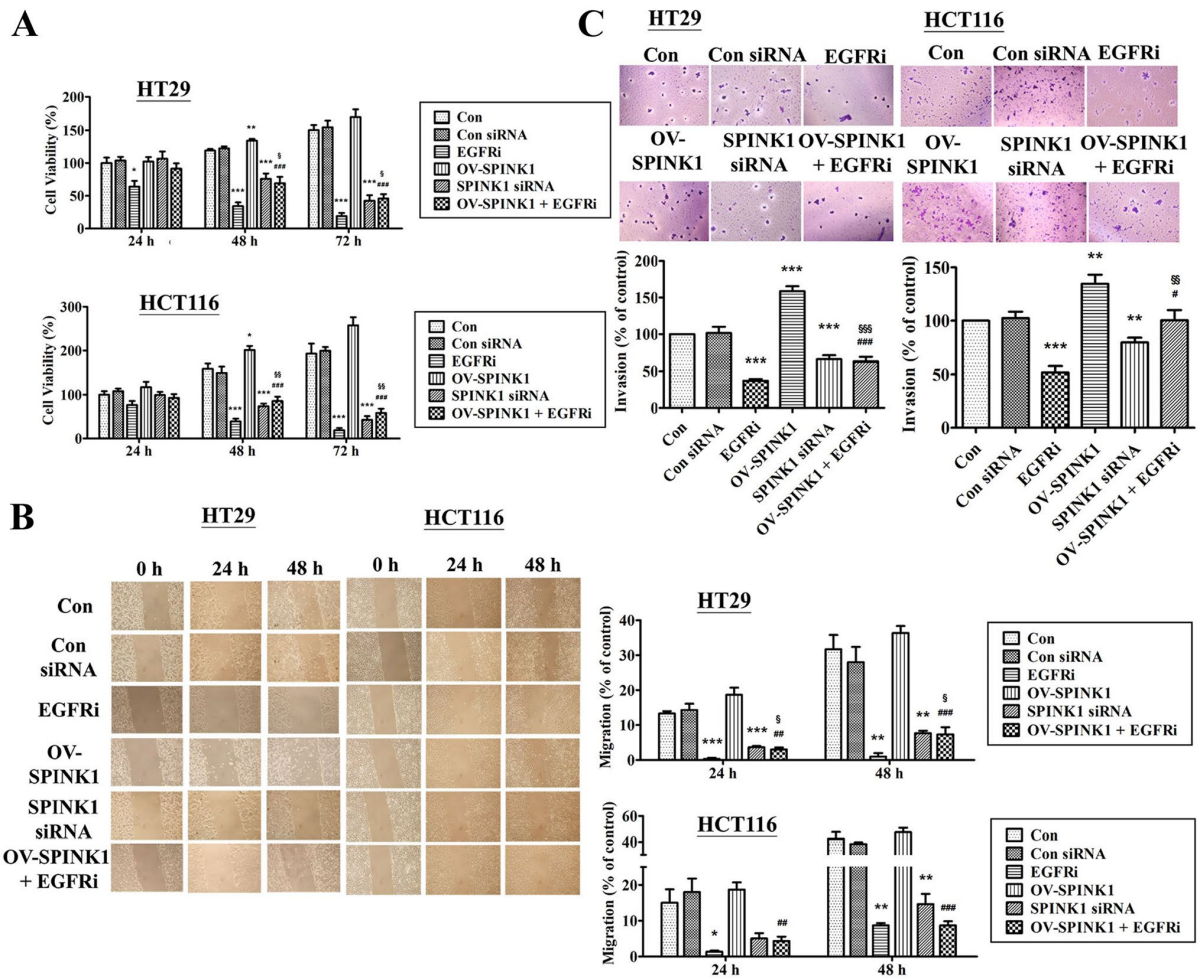
**A** The growth curve of HT-29 and HCT-116 cells transfected with non-siRNA, EGFR inhibitor, p-SPINK1 and SPINK1 siRNA (with/without EGFR inhibitor) cultured in 24 well plates during three days. **B** Wound healing assay and the percentage of migration cells of HT-29 and HCT-116 cells in 0 h, 24 h and 48 h after being treated with non-siRNA, EGFR inhibitor, p-SPINK1 and SPINK1 siRNA (with/without EGFR inhibitor). **C** Transwell invasion assay and the number of invaded cells of HT-29 and HCT-116 cell lines during a single day transfected treatment with non-siRNA, EGFR inhibitor, p-SPINK1 and SPINK1 siRNA (with/without EGFR inhibitor). The quantitative data analysis was expressed as mean ± SEM. *p < 0.05, **p < 0.01 and ***p < 0.001 as compared to the control group (n = 3/group); ^#^*p* < 0.05, ^##^*p* < 0.01 and ^###^*p* < 0.001 as compared to the OV-SPINK1 group; ^§^*p* < 0.05, ^§§^*p* < 0.01§§ and ^§^*p* < 0.001 as compared to the EGFRi group (*n* = 3/group)

To study the capacity of proliferation of CRC regulated by SPINK1, we compared the cell numbers between HT-29 and HCT-116 with si-SPINK1 or non-siRNA. After 72 h of incubation with siRNA, the cell viability was assayed by XTT assay. In both CRC cell lines, the results indicated that the cell viability of the si-SPINK1 group was obviously attenuated compared with that in the control group (*p* < 0.001). No differences were found between control and non-siRNA groups. Furthermore, SPINK1 expression vector was transfected into HT-29 and HCT-116 cells, and the number of cells was also significantly increased under the effect of endogenous SPINK1 up-regulation in HCT 116 cells (*p* < 0.01) (Fig. 3A).

On the other hand, to evaluate the influence of EGFR inhibitor on cell proliferation in CRC cells, HT-29 and HCT-116 cells with OV-SPINK1 were treated by EGFR inhibitor. The data showed that EGFR inhibitor was able to inhibit proliferation in these cells with hyper-endogenous SPINK1 and the effect of EGFR inhibitor was also reversed by hyper-endogenous SPINK1 (Fig. 3A).

These results implied that the proliferation of CRC cells was regulated by knock-down of SPINK1, through adding EGFR inhibitor or recombinant SPINK1.

### SPINK1 up-regulation promotes CRC cell migration and invasion

To demonstrate the abilities of migration and invasion in CRC cells, we used wound healing and transwell assay to analyze the mechanism in vitro. A knock-down of SPINK1 expression with specific SPINK1 siRNA markedly inhibited the capability of migration (Fig. 3B) and invasion in both HT-29 and HCT-116 cell lines (Fig. 3C).

Furthermore, we transfected SPINK1 expression vector into cells, and the number of transfected cells with OV-SPINK1 tended to increase the ability of migration in both cell lines; furthermore, OV-SPINK1 promoted the invasion in both HT-29 and HCT-116 cells.

Additionally, to investigate the interaction of EGFR and SPINK1 on cell migration and invasion in CRC cells, HT-29 and HCT-116 cells with OV-SPINK1 were also treated by EGFR inhibitor. The data revealed that EGFR inhibitor was able to inhibit the migration and invasion in CRC cells with hyper-expressed SPINK1 (*p* < 0.001).

Overall, the results demonstrate that SPINK1 plays an important role for promoting cell ability of migration and invasion, and is mediated through the EGFR pathway in CRC cells.

### SPINK1 interact with EGFR and co-expression of SPINK1 and EGFR

**Fig. 4:**
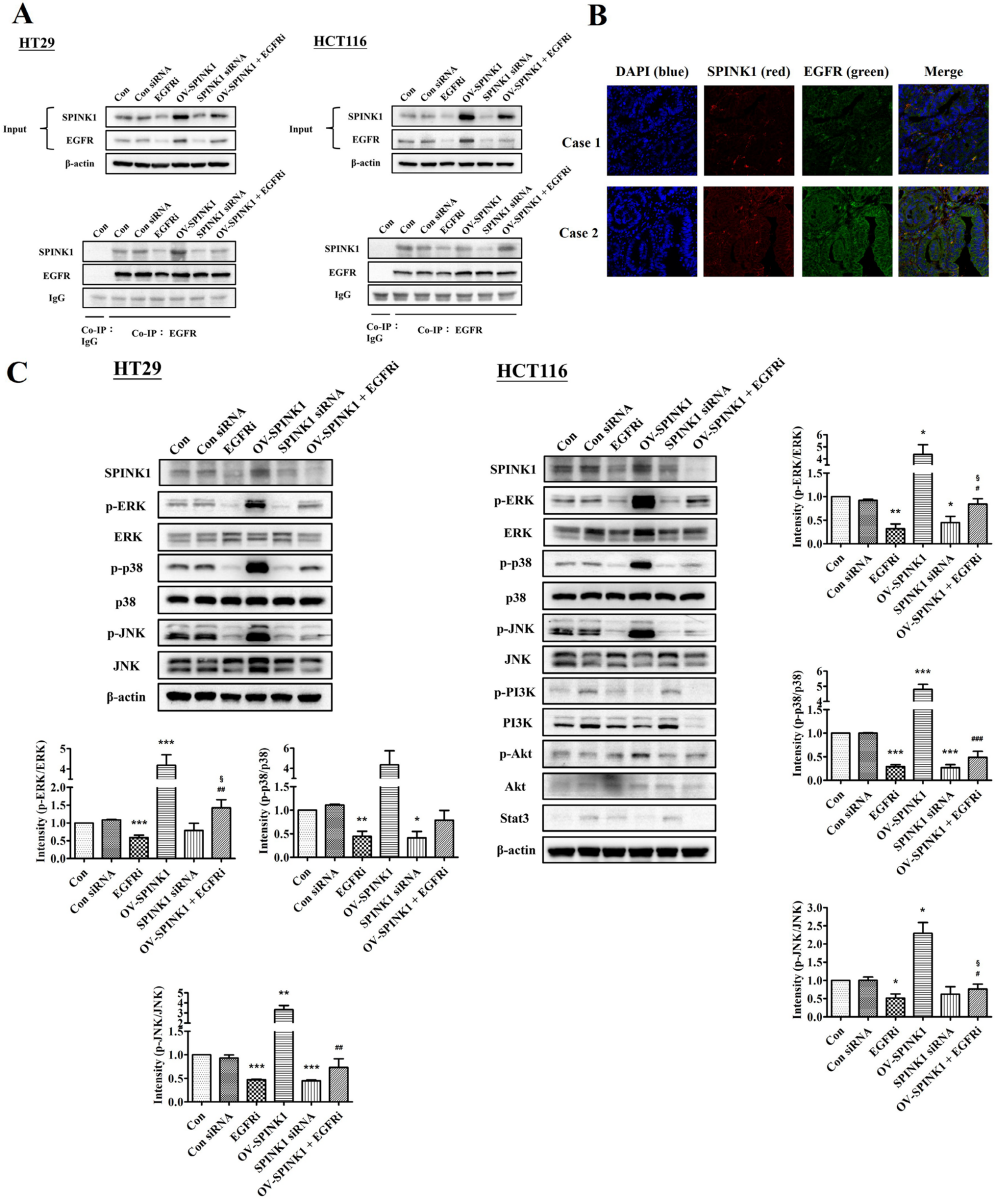
**A** SPINK1 directly interacts with EGFR. Cell lysates from HT-29 and HCT-116 cells treated with non-siRNA, EGFR inhibitor, p-SPINK1 and SPINK1 siRNA (with/without EGFR inhibitor) were immunoprecipitated with the EGFR antibody, followed by immunoblotting with SPINK1 antibodies. **B** Immunofluorescence visualization of Co-expression of SPINK1 and EGFR was observed in representative specimens of colorectal cancer patients. To visualize the cell nuclei, the cells were mounted with a DAPI-containing mounting medium (blue). SPINK1 was detected using the anti-SPINK1 antibody and a FAM-conjugated secondary antibody (red). EGFR was detected using the anti-EGFR antibody and TAMRA-conjugated secondary antibody (green). All channels merged. Objective 20x. (C) Western blot analysis for p-ERK, ERK, p-p38, p38, p-JNK, JNK and SPINK1 levels transfected with non-siRNA, EGFR inhibitor, p-SPINK1 and SPINK1 siRNA (with/without EGFR inhibitor), while a loading control β-actin was used. The quantitative data analysis was expressed as mean ± SEM. *p < 0.05, **p < 0.01 and ***p < 0.001 as compared to the control group (*n* = 3/group); ^#^*p* < 0.05, ^##^*p* < 0.01 and ^###^*p* < 0.001 as compared to the OV-SPINK1 group. ^§^*p* < 0.05 as compared to the EGFRi group (*n* = 3/group)

According to previous results and the literature, the interaction between SPINK1 and EGFR seemed to be a fetal pathway to regulate CRC pathogenesis. Taking this into account, co-immunoprecipitation and immunofluorescence experiments of SPINK1 and EGFR were also performed. Importantly, immunoprecipitation of EGFR resulted in the identification of SPINK1 in all different groups, demonstrating the coexistence of both proteins in CRC cell lines (Fig. 4A). The immunofluorescence assay showed co-expression of SPINK1 (red) and EGFR (green) in CRC patients specimens (Fig. 4B).

### SPINK1 promotes CRC cell lines proliferation, migration and invasion via ERK, p38, and JNK pathways

To clarify the signaling pathway of SPINK1 through EGFR to regulate CRC cells, western blot analysis of EGFR downstream proteins was performed in vitro. The results showed that higher expression of p-ERK, p-p38 and p-JNK were noted in cells with OV-SPINK1, combined with lower expression of p-ERK, p-p38 and p-JNK in transfected SPINK1 cells. Also, pERK p-p38 and p-JNK were decreased in SPINK1 siRNA cells compared to control cells. No significant change of PI3K/Akt or Stat3 was identified in these cells (Fig. 4C).

**Fig. 5:**
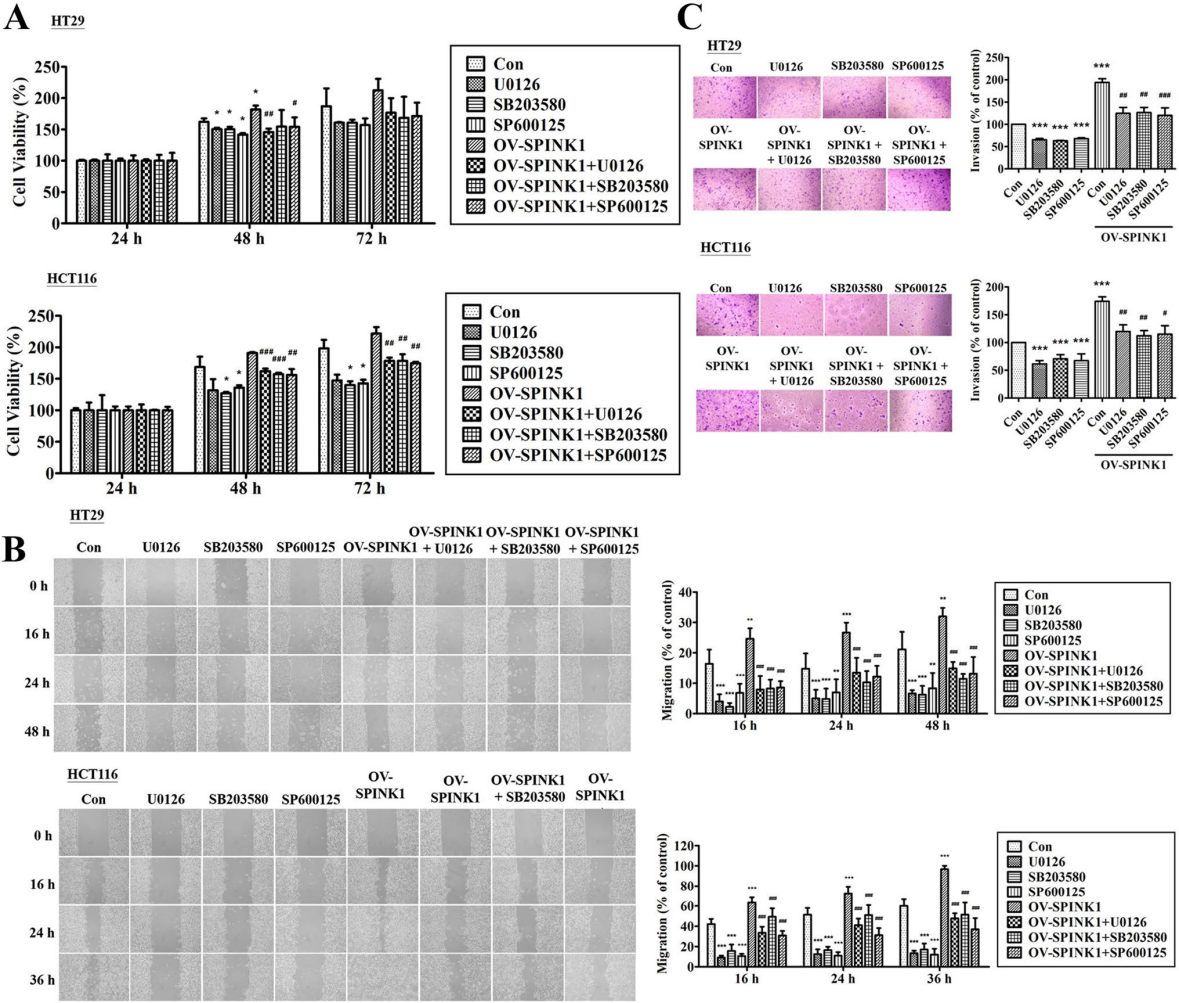
**A** The growth curve of HT-29 and HCT-116 cells treated with p-SPINK1 and inhibitors cultured in 24 well plates in 24 h, 48 h and 72 h. **B** Wound healing assay and the percentage of migration cells of HT-29 and HCT-116 cells in 0 h, 24 h, 36 h and 48 h after treated with p-SPINK1 and inhibitors. **C** Transwell invasion assay and the number of invaded cells of HT-29 and HCT-116 cell lines during one day transfected treatment with p-SPINK1 and inhibitors. The quantitative data analysis was expressed as mean ± SEM. *p < 0.05, **p < 0.01 and ***p < 0.001 as compared to the control group (*n* = 3/group); ^#^*p* < 0.05, ^##^*p* < 0.01 and ^###^*p* < 0.001 as compared to the OV-SPINK1 group (*n* = 3/group)

To analyze which pathway was involved in cell biology, variant inhibitors were added in CRC cell lines. When HC-29 and HCT-116 cells were treated with the ERK inhibitor (U0126), p38 inhibitor (SB203580) and JNK inhibitor (SP600125), cell numbers tended to decrease compared to those without inhibitors (Fig. 5A). Migration and invasion of CRC cell lines were also inhibited by ERK, p38 and JNK inhibitors (Fig. 5B, C).

### SPINK1 regulates radioresistance in CRC cell lines through ERK, p38, and JNK pathways

**Fig. 6:**
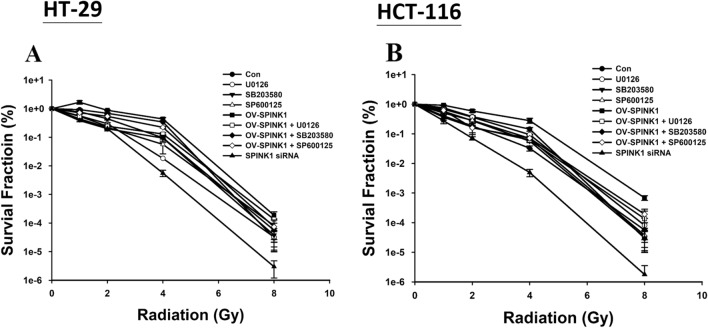
Effect of silencing SPINK1, OV-SPINK1 and ionizing radiation on colony formation in colon cancer cells treated with ERK, p38 or JNK inhibitor (*n* = 3/group) **A** HT-29 (control vs. siRNA group, *p* < 0.001; control vs. OV-SPINK1 group, *p* < 0.001; OV-SPINK1 vs. OV-SPINK1 + U0126, *p* < 0.001; OV-SPINK1 vs. OV-SPINK1 + SB203580, *p* < 0.001; OV-SPINK1 vs. OV-SPINK1 + SP600125, *p* < 0.001) and **B** HCT-116 (control v.s. siRNA group, *p p* < 0.001; control vs. OV-SPINK1 group, *p* < 0.001; OV-SPINK1 vs. OV-SPINK1 + U0126, *p* < 0.001; OV-SPINK1 vs. OV-SPINK1 + SB203580, *p* < 0.001; OV-SPINK1 vs. OV-SPINK1 + SP600125, *p* < 0.001) cell lines

To identify the radio-sensitizing effect and the associated pathway of SPINK1, CRC cell lines HT-29 and HCT-116 with or without SPINK1 knockdown treated with the ERK, p38 or JNK inhibitor were exposed to radiation at two different radiation doses (1 to 8 Gy). The clonogenic assay was performed showing that silencing SPINK1 could radio-sensitize colon cancer cell lines, and the combination of si-SPINK1 and radiation was superior to treatment by radiation alone (*p* < 0.001) (Fig. 6). Moreover, transfected cells with OV-SPINK1 increased radioresistance compared with the control group (*p* < 0.001). The effects were reversed after treatment by ERK, p38 or JNK inhibitor (all *p* < 0.001) in both cell lines.

## Discussion

SPINK1 is a 6 kDa polypeptide, encoded by *SPINK1* gene located at 5q32, and is also known as pancreatic secretory trypsin inhibitor or tumor-associated trypsin inhibitor. It functions as a trypsin inhibitor mainly in the pancreas, preventing self-digestion in pancreatic cells, and it exhibits a fetal role in chronic pancreatitis [4]. An experiment involving pancreatic acinar cells of mice demonstrated that SPINK1 inhibited autophagy while maintaining the integrity and regeneration of acinar cells [19]. In liver cells infected by Hepatitis B and C, SPINK1 also protected hepatocytes from cellular apoptosis [20]. SPINK1 has been found in various cell lines and is involved in healing response after injury [21]. Overall, SPINK1 appears to have a potential function in cellular survival.

The association of SPINK1 and carcinogenesis has also been established. With the exception of gastric malignancy, poor prognosis is found in patients with high SPINK1 expression, including those of pancreas, prostate, ovary, breast, liver, lung and colorectal cancers [6, 22–27]. SPINK1 overexpression is not only caused by TMPRSS2:ERG chromosomal rearrangement, deletion, or amplification, but also by posttranscriptional modification [28]. Additional evidence shows that inducing autocrine and paracrine formation may disrupt the balance of cell–matrix interactions to enhance cancer invasiveness and metastasis [29]. The function of SPINK1 may not only be mediated by a serine protease inhibitor but also through alternative mechanisms of action. In estrogen-receptor positive breast cancer, SPINK1 can promote invasiveness in addition to resistance to drug-induced apoptosis [25]. Lee et al. demonstrated that SPINK1 exhibits a potential stepwise driver gene of hepatocellular carcinoma by ER stress-associated epigenetic DNA methylation [30]. Loss of SPINK1 attenuates invasiveness and tumor growth as has been noted in prostate cancer [11]. In a SPINK1-positive prostate cancer mouse model, cancer-associated mortalities were confirmed and miRNA-338-5p/-421 abrogated oncogenic effects including cell-cycle progression, stemness, metastasis and drug resistance [31]. Knockdown of SPINK1 suppressed the peritoneal metastasis of ovarian clear cell carcinoma in an animal experiment [32]. These findings suggest the oncogenic potential and therapeutic target of SPINK1.

Mechanically, because of molecular structural similarity with EGF, the association between SPINK1 and EGFR has been studied in many cancers. SPINK1 caused epithelial-endothelial transition mediated by EGFR signaling [29]. In a SPINK1-positive prostate cancer xenografted mice study, monoclonal EGFR antibody was administered and showed decrease in tumor burden, indicating interaction with EGFR [11]. Ozaki et al. demonstrated that SPINK1 could bind to EGFR to induce cell proliferation and that it was mediated by the MAPK/ERK pathway in the pancreas [10]. SPINK1 overexpression was noted to increase cell migration and invasion associated with epithelial-mesenchymal transition via MAPK and the extracellular-regulated kinase pathway in hepatocellular carcinoma [30].

This study performed in vitro experiments to evaluate SPINK1 effects in CRC cell progression. SPINK1 transcript levels were attenuated by siRNA and overexpression in CRC cell lines. Assays were conducted to detect phenotypic changes in cell proliferation, migration and invasiveness, in line with a previous result [33]. EGFR inhibitor significantly decreased tumor ability of proliferation, migration and invasion, rescued by upregulation of endogenous SPINK1. In previous reports, HT-29 cell line was found to be highly expressive in secreting SPINK1. It mediated autocrine invasion and metastasis and was negatively regulated by anti-SPINK1 antibody and function-blocking mutant *KY-SPINK1* at the serine -protease interaction site involving adenoma-carcinoma transition [34]. The addition of exogenous SPINK1 stimulated HT-29 cell migration, but the effect was reduced after application of EGFR blocking antibody in wound monolayer analysis [35]. Downstream activation of phosphatidylinositol 3-kinase and MAPK/ERK pathways by SPINK1 was noted in CRC cell lines harboring *KRAS*, *NRAS* or *BRAF* mutations by Tiwari et al. [33].

The impact on cell proliferation and carcinogenesis of SPINK1 by silencing SPINK1 or transfected SPINK1 expression in carcinogen-induced colitis-associated cancer was also evaluated in a mouse model [36]. In our findings, not only MAPK/ERK pathway but also p38 and JNK signaling influenced downstream SPINK1 to regulate cellular proliferation, migration and invasion proven by adding different inhibitors. Chang et al. showed that SPINK1 promoted rat hepatocyte proliferation via p38, ERK and JNK pathways in vitro [37]. In prostate cancer, miR-5089-5p suppressed SPINK1 mRNA by binding to its 3’UTR to up-regulate matrix metalloproteases 9 and reversed the effect of proliferation, migration and metastasis in drug-resistant cells [38]. The results above illustrate that SPINK1 could combine with EGFR to regulate various carcinogenic types via ERK, p38 and JNK pathways and that may be an oncogenic driver of aggressiveness in CRC.

The chi-square analysis in our study showed that high pre-CCRT SPINK1 expression was significantly associated with PNI and poor CCRT response. Furthermore, high post-CCRT SPINK1 expression was significantly associated with advanced pathological cancer stage and poor CCRT response, which is in line with the previous literature [5, 36]. Kaplan–Meier analysis of the RC patients revealed significantly shorter OS and DFS in those with high SPINK1 expression for both pre- and post-CCRT status, in line with another study [12]. Aggressiveness and recurrence were also correlated with high SPINK1 expression in other cancers [38]. Multivariate analysis of OS showed that disease stage and pre-CCRT SPINK1 expression was significantly associated with the prognoses of RC patients, while pre-CCRT SPINK1 expression and metastasis had independent prognostic roles for DFS. Overall, pre-CCRT SPINK1 is a pivotal biomarker for the prognosis of RC receiving pre-operative CCRT.

Radiation induces DNA damage and apoptotic cell death in epithelial cells as well as in endothelial cells supplying blood vessels through the intrinsic pathway via mitochondria [39]; however, cancer cells have the capacity to evade apoptosis. SPINK1 was found to reduce hypoxia-induced cell death in human gastric and colonic cells [40]. Anti-apoptotic effect of SPINK1 was observed in breast and lung cancers, as a caspase independent innate apoptotic pathway [25, 41, 42]. SPINK1 reducing pro-apoptotic caspases 3, 9 and Bax α levels combined with normalizing Bcl-2 were observed in the gut of mice [40]. In BRL-3A hepatocellular carcinoma cells, upregulation of endogenous or exogenous recombinant SPINK1 was noted to be associated with the effect of anti-apoptosis [37]. In non-small cell lung cancer, inhibition of SPINK1 was found to increase reactive oxidative species production and tumor cell apoptosis mediated by the p38 MAPK signaling pathway [42]. Attenuation of SPINK1 expression in CRC may promote the efficacy of radiotherapy by decreasing effects of anti-apoptosis.

Overall, this study has several limitations. Firstly, some clinical data such as surgical methods and chemotherapy regimens were precluded due to the retrospective design; however, all patients were selected at a single academic medical center, which strengthens the findings. Secondly, the reliability of IHC procedures might be affected by the fixation technique, clone of the antibody, antibody concentration, and interpretation criteria. Further prospective study designs combined with animal models is needed for further investigation of SPINK1 as a biomarker used in targeted therapy.

## Conclusion

This study suggests that SPINK1 expression is an independent prognostic marker in patients receiving pre-operative CCRT. SPINK1 regulates CRC proliferation, migration and invasion through ERK, p38 and JNK pathways, leading to poor clinical outcomes. The phenotypes of radiosensitivity able to be reversed with attenuation of SPINK1 levels suggest that SPINK1 could be a potential therapeutic target for precision medicine.
